# The Cannabis Health Literacy Questionnaire – Assessing Reliability and Known-Groups Validity in a Canadian Adult Sample

**DOI:** 10.1177/00472379261439959

**Published:** 2026-04-08

**Authors:** Queen Jacques, Zhiwei Gao, Maisam Najafizada, Lisa Bishop, Rachel Howells, Jennifer Donnan

**Affiliations:** 1 Division of Population Health and Applied Health Sciences, Faculty of Medicine, 7512 Memorial University of Newfoundland, St. John's, Canada; 2 School of Pharmacy, 7512 Memorial University of Newfoundland, St. John's, Canada

**Keywords:** Cannabis health literacy, cannabis, health literacy, measurement, drug education

## Abstract

This study assessed the reliability and known-groups validity of the Cannabis Health Literacy Questionnaire (CHLQ) in a sample of Canadian adults (19+). A total of 1,034 respondents completed the CHLQ online. The CHLQ comprises four dimensions: Knowledge of Cannabis, Knowledge of Risks, Understanding Harms and Risks, and Seeking, Accessing and Using Cannabis Health Information. Internal consistency was assessed with raw questionnaire scores, while known-groups validity was assessed using Rasch-derived person scores with one-way analyses of variance. Internal consistency was acceptable for the Likert-response dimensions (Cronbach's α: 0.76–0.84) and modest for the knowledge-based dimensions (KR-20: 0.59 −0.64). CHLQ scores varied across participant characteristics, with cannabis consumption status emerging as a key characteristic across dimensions. Current cannabis consumers demonstrated higher knowledge of cannabis and confidence in seeking and using cannabis health information but lower agreement with cannabis risk related statements. Age-related differences were also observed, with younger and middle-aged adults scoring higher than older adults in select CHLQ dimensions. These findings provide additional evidence supporting the CHLQ's reliability and construct validity and demonstrating its potential use for evaluating variation in cannabis health literacy across population subgroups. Such evidence may help informed future intended uses of the CHLQ and efforts to refine public-oriented cannabis health education.

## Introduction

Cannabis use, whether for medicinal or recreational purposes, can have some potential health effects on an individual's body and brain (CDC, 2024; Sachs et al., 2015). These potential effects arise from psychoactive and non-psychoactive compounds in the cannabis plant (i.e., cannabinoids), such as Δ9-tetrahydrocannabinol (THC) and cannabidiol (CBD), which act on neurobiological systems and shape how individuals experience cannabis (Cohen et al., 2019; Health Canada, 2018a). While cannabis may be used for therapeutic and recreational purposes, both short-term and long-term adverse effects have been documented, and the magnitude of potential harms varies by individual and contexts of use (Hall & Degenhardt, 2009; Health Canada, 2018a, 2022).

Evidence-informed health risks with cannabis use include the potential for cannabis dependence, adverse mental and physical health outcomes, and cognitive implications, particularly when use begins at a younger age (Fischer et al., 2017; Hoch et al., 2015; National Academies of Sciences et al., 2017). Short-term adverse effects include impaired driving, panic attacks and acute psychiatric episodes, whereas long-term adverse effects may include respiratory illness, cannabis hyperemesis syndrome, dependence and worsening of mental health conditions (Cohen et al., 2019; Fischer et al., 2017; National Academies of Sciences et al., 2017). At the same time, some studies report potential therapeutic benefits of cannabis for specific health conditions such as anxiety, cancer symptom management, pain associated with HIV/AIDS and arthritis, and some individuals self-report perceived benefits to health and well-being (Cohen et al., 2019; Holden et al., 2022; Paland et al., 2023; Tripp et al., 2014; Woolridge et al., 2005). Although adverse effects are supported by evidence, the literature remains heterogeneous, and the severity and likelihood of potential benefits and harms are still limited and vary by patterns and amount of cannabis use (Hoch et al., 2025; Solmi et al., 2023). Based on the current evidence, public-oriented health education about potential cannabis-related risks remains an important public health priority in Canada consistent with the goals of legalization. In this context, public-oriented health education refers to population-level communication efforts such as public health campaigns, product labeling with warnings and informational resources designed to support informed decision making, rather than school-based curricula.

While some studies have explored perceptions and attitudes toward cannabis (Bayat et al., 2023; Colonna et al., 2021; Hallett & Chen, 2023; Kleidon et al., 2023), fewer have measured public knowledge of adverse cannabis health effects in depth. Addressing this gap requires evaluating not only campaign reach but also how well people access, understand and apply cannabis-related health information, a concept defined as Cannabis Health Literacy (CHL). CHL is the degree to which individuals can seek, understand, and apply cannabis health information to make safe and informed choices regarding cannabis use (Jacques et al., 2025). Evaluating CHL can help identify knowledge gaps, inform targeted educational initiatives and support evidence-based public health strategies.

To measure CHL, the Cannabis Health Literacy Questionnaire (CHLQ) was developed and preliminarily validated to assess knowledge and skills aligned with evidence-informed information disseminated after cannabis legalization in Canada. The CHLQ is grounded in Nutbeam's health literacy framework, which emphasizes functional (i.e., ability to read, understand and use health information) and interactive (i.e., skills allowing individuals to engage and apply information) health literacy (Berkman et al., 2010; Jacques et al., 2025; Nutbeam, 2008). These domains reflect competencies used to navigate the complexities of cannabis-related health information, though individuals’ knowledge and skill may vary by context.

Population differences in general health literacy are well documented across sociodemographic factors such as age, income and ethnicity (HLS-EU Consortium, 2012; Klinker et al., 2020; Liu et al., 2015; Rolova et al., 2020; Sentell & Braun, 2012). In parallel, national data from the Canadian Cannabis Survey (CCS) demonstrate that patterns of cannabis use and associated health risks vary across sociodemographic groups. For example, 26% of Canadians aged 16 years and older reported used cannabis for non-medical purposes in the past 12 months (Health Canada, 2024c), with higher use among individuals aged 20–24 (48%) and among males (28% vs. 23% among females). Cannabis use was also higher among individuals with less than high school or a high school diploma (32%) compared to those with postsecondary education (Health Canada, 2024c). The Lower-Risk Cannabis Use Guidelines (LRCUG) further highlights higher health risks from cannabis consumption among youth, individuals with lower socioeconomic status, and those with a history of substance use (Fischer et al., 2022). Together, these subgroup differences provide rationale to examine CHL across sociodemographic groups and assessing how the CHLQ captures this variation.

Building on the initial validation of the CHLQ, which demonstrated sound measurement properties and construct validity, this study extends validation by examining the tool's reliability and known-groups validity across sociodemographic subgroups with the same Canadian adult sample. Consistent with best practices in test development outlined in the *Standards for Educational and Psychological Testing* (American Psychological Association et al., 2014), we assess whether the CHLQ performs consistently across relevant subgroups and whether it detects theoretical difference in CHL. We use sociodemographic (e.g., age, sex and education) and cannabis use characteristics as a priori known-group comparisons and evaluate group differences in an exploratory, descriptive manner. By clarifying subgroup variation in the CHLQ, the study aims to (1) strengthen the interpretability and intended use of the CHLQ, and (2) support future research examining whether subgroup differences in CHL may be relevant to public health communication and education.

## Methods

### Study Design

This cross-sectional study was conducted across Canada in March 2023 through an online survey. Respondents completed an anonymous survey assessing general health status, cannabis consumption history, CHL, and sociodemographic characteristics. The full cross-sectional survey is provided in Appendix A.

### Participants and Procedure

Data from participants who contributed to the finalized version of the CHLQ, as reported in Jacques et al. (2025), were used in the present study. Survey respondents were recruited through the Angus Reid Forum^©^, an online platform for Canadian subscribers, who are invited via email to complete surveys based on a variety of topics (Angus Reid, 2023). Available study funding supported the distribution of the survey through this platform.

Adults aged 19 and older residing in Canada were recruited. The survey was administered on Qualtrics^©^, a web-based survey platform, and shared through Angus Reid until a target of 1,000 eligible respondents completed the survey. A target sample size of 1,000 participants was selected to support the initial psychometric development and evaluation of the CHLQ, including stable item parameter estimation in Rasch analyses (Boone et al., 2014; Linacre, 1994).

To ensure participants anonymity, no personal identifying information was collected (i.e., names or contact information). To ensure data quality and prevent duplicate or fraudulent responses, built-in Fraud Detection tools (i.e., IP address checks, bot detection and response speeds) provided by Qualtrics were enabled (Qualtrics XM, 2023).

### Measures

#### Demographic Data

Demographic information collected included age, biological sex, gender, education level, and provincial location (i.e., province or territory of residence). Age groups were displayed in the survey as 10-year ranges (i.e., 19–29). We further re-grouped them into three categories ‘adults (19–39 years old)’, ‘middle aged adults (40–59 years)’ and ‘older adults (60–70 + years old)’, grounded in evidence that individuals across these age ranges exhibit varying interactions with cannabis-related information and may be influenced by their unique life experiences and societal contexts (Carliner et al., 2017; Haug et al., 2017; MacDougall & Maston, 2023).

Biological sex (i.e., sex determined at birth) was categorized as ‘male’, and ‘female.’ Gender, a social construct of roles, behaviors and expression of individuals, was grouped into four categories; ‘Man’, ‘Woman’, ‘Gender diverse’ and ‘Prefer not to say’. Gender was omitted from the analysis due to collinearity with biological sex in our study population (Gender VIF = 24.31, Biological Sex VIF = 45.14). The collinearity indicated that the two variables were highly correlated and provided redundant information.

Education levels were grouped into four categories based on the highest level of education attained: ‘High school’ (i.e., some high school attend and/or high school diploma), ‘Undergraduate/College’ (i.e., some university/college attended and/or bachelor's degree or college diploma), ‘Graduate degree’ (i.e., master's or doctoral degree), ‘Professional degree’ (i.e., Doctor of Medicine, Doctor of Dental Surgery, Doctor of Veterinary Medicine, etc.). Lastly, provincial location within Canada, defined by the province or territory in which individuals reside, was grouped into five categories based on the Canadian regions (Immigration, 2009): ‘Atlantic’, ‘Central’, ‘Prairies’, ‘West Coast’ and ‘North.’

#### Cannabis Consumption status

Respondents were asked to report their cannabis consumption using response options that included ‘I have never consumed cannabis’, ‘consuming prior to legalization’ and ‘started consuming post-legalization’. For the analysis, responses were regrouped into three categories representing never, past and current use. Participants who reported never having consumed were categorized as ‘never consumers.’ Participants who reported consuming prior to legalization but not currently consuming (i.e., in the past 12 months) were categorized as ‘past consumers.’ Current consumers were participants who reported consuming cannabis in the past 12 months, including those who consumed prior to legalization and continued, those who stopped and later restarted after legalization and those who initiated cannabis use after legalization.

#### Cannabis Health Literacy Questionnaire and Validation

Jacques et al. (2025) developed and preliminarily validated the CHLQ. The questionnaire was developed to measure an individual's knowledge, understanding, and utilization of health and safety cannabis information for research purposes. The CHLQ includes four scored dimensions, which served as our dependent variables in this study: (1) Knowledge of Cannabis, (2) Knowledge of Risks, (3) Understanding Harms and Risks, and (4) Seeking, Accessing, and Using Cannabis Health Information. Table 1 describes each dimension with the number of questions and their response and coding formats. ‘I don’t know’ responses for any of the questions were treated as incorrect.

**Table 1.** Overview of CHLQ Dimensions and Measurement Characteristics.

CHLQ Dimensions	Description	Number of Questions	Response Format	Coding
1. Knowledge of Cannabis	General Knowledge (e.g., reading labels, potency, THC/CBD)	8	Multiple-choice	Correct or Incorrect *I don’t know answers were coded as incorrect
2. Knowledge of Risks	Awareness of physical & mental health risks	6	5-point agreement Likert scale	Likert scores
3. Understanding Harms and Risks	Knowledge of short- and long-term harms	6	Multiple-choice	Correct or Incorrect *I don’t know answers were coded as incorrect
4. Seeking, Accessing, and Using Cannabis Health Information	Ability to find and use cannabis related information	4	5-point agreement Likert scale	Likert scores

Rasch analysis (via WINSTEPS v.5.3.3.1) was used to validate the CHLQ and assess item difficulty, fit, and measurement properties (Boone et al., 2014; Jacques et al., 2025). Each dimension of the CHLQ demonstrated unidimensionality and an acceptable range of item difficulties, with moderate to high item separation and reliability values described in the initial validation study (Jacques et al., 2025). Rasch modeling transforms raw scores into linear logits (log-odds units) scores (Andrich & Marais, 2019; Boone, 2016) and were used to examine known-groups validity, as the CHLQ was originally developed and calibrated within a Rasch measurement framework. Rasch estimates provide person scores adjusted for item difficulty, whereas are raw summed scores are ordinal and may not consider differences across levels of ability and item difficulty (Boone et al., 2014; Khan et al., 2015; Wu, 2007). A Rasch-score near 0 represents average performance within the sample, positive scores indicate above average ability (i.e., higher performance), and negative scores reflect below-average ability (i.e., low performance). The conversion of raw score to Rasch measurement scores for each question is relative to the sample within this study and is available upon request.

### Statistical Analysis

#### Descriptive Statistics

Descriptive statistics were used to summarize participants characteristics and the percentage of correct responses. Frequencies and percentages were calculated for categorical variables. For Likert-scale items, responses of “agree” and “strongly agree” were combined to report agreement. Multiple-choice items were coded as correct or incorrect, and the percentage of correct responses was analyzed. All analyses were performed using the Statistical Package for Social Sciences (SPSS v.30.0.1.1.).

#### Differential Item Functioning

Prior to our analysis, CHLQ item performance was assessed across groups to ensure fairness and minimize bias, given the tool's exploratory nature. Using the Rasch-Welch test and Mantel-Haenszel chi-square in WINSTEPS (v.5.3.3.1), we conducted differential item functioning (DIF) analyses focused on age and biological sex, which is commonly associated with DIF (Boone et al., 2014; Bourion-Bédès et al., 2015; Teresi & Fleishman, 2007; Walker, 2011; Yadegari et al., 2019). Items showing significant DIF (*p* < 0.001) with moderate to large effect sizes (> 0.64) were flagged. One item in the ‘Knowledge of Cannabis’ dimension showed DIF by sex, with females finding it easier to answer correctly. However, per Rasch guidelines, DIF alone doesn't indicate bias. We retained the item after further examining response distributions (Appendix B).

#### Reliability Analysis

Internal consistency reliability was performed for each CHLQ dimension. Raw CHLQ scores were used to test the reliability of the items within the dimensions. For domains using Likert scale responses (e.g., Knowledge of Risks and Seek, Access and Use of Cannabis Health Information), internal consistency was examined using Cronbach's alpha (α). For domains with dichotomous items scored as incorrect or correct (e.g., Knowledge of Cannabis and Understanding Harms and Risks), internal consistency was assessed using the Kuder-Richardson Formula (KR-20) (Nugroho et al., 2019; Schober et al., 2018).

Item-total correlations were examined to evaluate item contribution to their respective dimension. Listwise deletion was applied for missing responses. Reliability estimates were interpreted as indicators of internal consistency within the context of a new developed measure, with Cronbach's α/KR-20 values around 0.70 and above considered acceptable for Likert-type dimensions (Schober et al., 2018), and values ≥0.50 considered moderate and acceptable for knowledge-based dichotomous dimensions (Nugroho et al., 2019; Schober et al., 2018).

#### Known-Groups Validity Analysis

Known-groups validity was examined using the Rasch-derived person scores. Grouping variables were selected based on a priori theory and existing empirical literature that states cannabis exposure and demographic characteristics may contribute to differences in access to, engagement with and interpretation of cannabis-related information (Fischer et al., 2017; Health Canada, 2024c; Rolova et al., 2021). Specifically, age group, biological sex, education level, cannabis consumption status and provincial region were selected variables based on the national surveillance data from the CCS (Health Canada, 2024c). These variables were selected to evaluate if the CHLQ could differentiate between groups that may vary in CHL.

Group differences for each CHLQ dimension were assessed using one-way analyses of variance (ANOVA), with each variable assessed separately. Participants with missing demographic data were excluded from analyses for those variables. Prior to conducting the ANOVA, the assumption of homogeneity of variance was evaluated using Levene's test. When the assumption was met, one-way ANOVA was used. When homogeneity of variance was violated, Welch's ANOVA was conducted as a robust alternative. Statistical significance was set at *p* < 0.05. Effect sizes were also recorded using eta-squared (η^2^). Where ANOVA tests were statistically significant, post-hoc pairwise comparisons were conducted using Tukey's Honestly Significant Difference (HSD) to identify specific group differences. Sensitivity analysis was also conducted with supplementary ANCOVA models (general linear model, univariate) adjusting for cannabis consumption status to examine whether observed group differences were robust across the sociodemographic factors explored.

## Results

### Respondent Characteristics

We obtained responses from 1035 individuals across Canada, with one respondent being removed from analysis, as they did not complete the demographic section of the survey. Thus, the final analysis was conducted with 1034 respondents. Missing answers on the survey was minimal at 0.036%. Table 2 presents the demographic characteristics of our sample.

**Table 2.** Respondent Characteristics.

Variables	n (%)
Respondents	1034 (100)
Age	
Young Adults (19–39)	406 (39)
Middle aged adults (40–59)	328 (32)
Older adults (60–70+)	300 (29)
Biological Sex	
Female	525 (48)
Male	499 (51)
Prefer not to say	10 (1)
Cannabis Consumption Status	
Never consumed	335 (33)
Past consumer	269 (26)
Current consumer	428 (42)
Reason for consumption of cannabis	
Medical purposes (authorized or self-treated)	85 (23)
Recreational purposes (non-medical)	166 (45)
Both medical and recreational purposes	113 (31)
Education level	
Some HS/HS diploma	95 (9)
Bachelor/College degree	733 (71)
Graduate degree	160 (16)
Professional degree	45 (4)
Provincial regions*	
Atlantic (PEI, NS, NB, NL)	88 (9)
Central (QC, ON)	506 (49)
Prairie (MB, SK, AB)	255 (25)
West Coast (BC)	185 (18)
North (NWT, YK, NT)	0(0)

Note: PEI – Prince Edward Island; NS – Nova Scotia; NB – New Brunswick; NL – Newfoundland and Labrador; QC – Quebec; ON – Ontario; MB – Manitoba; SK – Saskatchewan; AB – Alberta; BC – British Colombia; NWT – Northwest Territories; YK – Yukon; NT – Nunavut; HS – High School.

Respondents correctly answered 61% of questions on the ‘Knowledge of Cannabis’ dimension, 82% on ‘Knowledge of Risks’ dimension, 48% on ‘Understanding Harms and Risks’ dimension and 75% on ‘Seek, Access and Use Cannabis Health Information’ dimension (Tables 3 and 4). Respondents found some questions challenging, where only 23% correctly answered the question, ‘*Too much of which ingredient in cannabis products can most likely lead to cannabis poisoning?*’ Similarly, 32% correctly answered the question, ‘*Which of the ingredients in cannabis products can give rise to adverse (i.e., unpleasant) side effects?*’ and 28% correctly responded to the question ‘*People can experience harm to brain development from cannabis use when they start using cannabis any time before the age of?*’.

**Table 3.** Percentage of Correct Responses in the CHLQ Dimensions: Knowledge of Cannabis and Understanding Harms and Risks (Using the Raw Survey Scores; Sample Size *N* = 1034).

CHLQ Dimensions	Question	Correct Responses (n, %)
**Knowledge of Cannabis (KC)**	According to the label displayed, what is the total THC in this product?	841 (81)
	According to the product label displayed below, how many milligrams (mg) of cannabinoids are in one soft gel?	697 (67)
	If one drop of CBD oil = 1.1.mg, how many drops would you need to have 16.5 mg of CBD?	908 (88)
	If a syringe of 1 mL has 20 mg of CBD, how many mL would you need to have 5 mg of CBD?	782 (76)
	Which of the ingredients of cannabis produces the feeling or experience of being “high”?	810 (78)
	Too much of which ingredient in cannabis products can most likely lead to cannabis poisoning?	234 (23)
	Which method of cannabis consumption typically has the longest delay before experiencing the feeling or experience of being high?	450 (44)
	Which of the ingredients in cannabis products is most likely to give rise to adverse (i.e., unpleasant) side effects?	334 (32)
	**Overall – KC dimension**	61%
**Understand Harms & Risks (UHR)**	After smoking cannabis, what is the minimum amount of time a person should wait before driving?	390 (38)
	People can experience harm to brain development from cannabis use when they start consuming cannabis any time before the age of?	284 (28)
	In your opinion, how common are hallucinations with THC consumption?	503 (49)
	In your opinion, how common are dry mouth/red eyes with THC consumption?	731 (71)
	In your opinion, how common is a rapid heart rate with THC consumption?	406 (39)
	In your opinion, how common is low appetite with THC consumption?	670 (65)
	**Overall – UHR dimension**	48%

**Table 4.** Percentage of Agreement Responses for the CHLQ Dimensions: Knowledge of Risks and Seek, Access, Use Cannabis Health Information (Using the Raw Survey Scores; Sample Size *N* = 1034).

CHLQ Dimensions	Question	Agreement Responses (n, %)
**Knowledge of Risks (KR)**	Smoking Cannabis can be harmful.	747 (72)
	Using cannabis when pregnant or breastfeeding can be harmful.	899 (87)
	Cannabis can be addictive.	725 (70)
	Driving or operating machinery after cannabis use is dangerous	951 (92)
	Regular cannabis use can increase the risk for psychosis or schizophrenia.	547(53)
	Teenagers are at a greater risk of harm from using cannabis than adults.	847 (82)
	**Overall – KR dimension**	82%
**Seek, Access and Use of Cannabis Health Information (SAU)**	I am confident I know where to find information about cannabis	832 (81)
	I am confident I can ask questions to a health care provider about cannabis.	617 (60)
	I am confident I know where to find information on how to manage unpleasant side effects with cannabis use.	613 (59)
	I am confident in using the cannabis information I find to make cannabis health-related decisions.	696 (67)
	**Overall – SAU dimension**	75%

### Reliability of the CHLQ Domains

Internal consistency reliability varied across the CHLQ domains (Table 5). The ‘Knowledge of Risks’ and ‘Seek, Access and Use of Cannabis Health Information’ dimensions demonstrated acceptable internal consistency (Cronbach's *α* = 0.76,0.84, respectively). The ‘Knowledge of Cannabis’ and ‘Understanding Harms and Risks’ dimensions demonstrated moderate internal consistency (KR-20 = 0.59, 0.64, respectively) (Schober et al., 2018). Item-total correlations were positive across all domains, indicating some level of meaningful contributions of items to their respective dimensions.

**Table 5.** Reliability of Cannabis Health Literacy Questionnaire Dimensions.

Dimensions	Items (n)	Item Type	Cronbach's α / KR-20	Corrected Item-Total Correlation Range
Knowledge of Cannabis	8	Dichotomous	0.59	0.25–0.39
Knowledge of Risks	6	Likert scale (5-point)	0.76	0.43–0.59
Understand Harms & Risks	6	Dichotomous	0.64	0.17–0.57
Seek, Access and Use of Cannabis Health Information	4	Likert scale (5-point)	0.84	0.52–0.75

### Known-Groups Validity (ANOVA Results)

#### Knowledge of Cannabis

The ‘Knowledge of Cannabis’ dimension assessed the ability to read and understand cannabis product labels, identify cannabis ingredients and evidence-informed adverse health effects. The scores in this dimension differed significantly by cannabis consumption status (F (2,645) = 53.40, *p* < .001, η^2^ = .097), age groups (F (2,666) = 14.06, *p* < .001, η^2^ = 0.029) and education level (F (3,1029) = 5.94, *p* < .001, η^2^ = .017) (Table 6). No significant differences were observed across biological sex or provincial region (*p* > .05). Post hoc comparisons indicated that current cannabis consumers scored higher than past and never consumers. Older adults scored significantly lower than both young adults and middle-aged adults. Additionally, individuals with some high school education or a high school diploma scored lower than those with a bachelor's, graduate or professional degree (Full post-hoc results are presented in Appendix C – Table 1). After adjusting for cannabis consumption status, age group and educational level differences remained statistically significant, biological sex and provincial region also remained not significant (Full ANCOVA results are presented in Appendix C – Table 2).

**Table 6.** Known-Groups Validity One-way ANOVA of Cannabis Health Literacy Questionnaire Dimensions.

Variables	F (df1, df2)	p	η^2^
*Knowledge of Cannabis Dimension*			
Age	14.06 (2,666) ^a^	**<0**.**001**	0.029
Biological Sex	0.23 (2, 1031)	0.792	0.000
Consumer Status	53.40 (2,645) ^a^	**<0**.**001**	0.097
Education level	5.94 (3,1029)	**<0**.**001**	0.017
Provincial region	1.47 (3, 1030)	0.221	0.004
*Knowledge of Risks Dimension*			
Age	0.96 (2,1031)	0.381	0.002
Biological Sex	0.94 (2,24) ^a^	0.360	0.002
Consumer Status	58.86 (2,604) ^a^	**<0**.**001**	0.101
Education level	2.44 (3,146) ^a^	0.067	0.008
Provincial region	0.42 (3,300) ^a^	0.743	0.001
*Understanding Harms and Risks Dimension*			
Age	44.77 (2,652) ^a^	**<0**.**001**	0.080
Biological Sex	0.57 (2,1031)	0.568	0.001
Consumer Status	114.65 (2,604) ^a^	**<0**.**001**	0.192
Education level	0.43 (3, 1029)	0.728	0.001
Provincial region	1.95 (3,1030)	0.120	0.006
*Seek, Access and Use Cannabis Health Information Dimension*			
Age	0.519 (2,1032)	0.595	0.001
Biological Sex	4.63 (2,1032)	**0**.**010**	0.009
Consumer Status	25.02 (2,1030)	**<0**.**001**	0.046
Education level	0.59 (3,1029)	0.669	0.002
Provincial region	2.40 (3,1031)	0.067	0.007

**Note.** Rasch-derived person scores were used. η^2^ = eta-squared.

^a^ Welch's ANOVA was used due to violation of homogeneity of variance.

#### Knowledge of Risks

‘Knowledge of Risks’ dimension measured an individual's agreement or disagreement with evidence-informed cannabis harms and risks statements. A significant difference in Knowledge of Risk scores was observed across cannabis consumption status (F (2,604) = 58.65, *p* < .001, η^2^ = .101). No significant differences were observed across age groups, biological sex, education level and provincial region (*p* > .05) (Table 6). Post hoc comparisons indicated that current cannabis consumers significantly scored lower than past or never consumers (*p* < .001). After adjusting for cannabis consumption status, all other variables remained not significant.

#### Understanding Harms and Risks

The ‘Understanding Harms and Risks’ dimension measured an individuals’ ability to interpret and understand evidence-informed adverse harms and risks with cannabis use. Scores in this dimension differed significantly across age groups (F (2,652) = 44.77, *p* < .001, η^2^ = .080), and cannabis consumption status (F (2,604) = 114.65, *p* < .001, η^2^ = 0.192) (Table 6). Post hoc comparisons demonstrated that older adults scored significantly lower than young and middle-aged adults. Additionally, current cannabis consumers scored significantly higher than past and never consumers. After adjusting for cannabis consumption status, age group differences remained statistically significant.

#### Seek, Access and use Cannabis Health Information

The ‘Seek, Access and Use Cannabis Health Information’ dimension measured individuals’ agreement or disagreement with their ability to seek, access and use evidence-informed cannabis information to make safe-informed decisions. Scores in this dimension differed significantly by biological sex (F (1,1032) = 4.63, *p* = .010, η^2^ = .009) and cannabis consumption status (F (2,1030) = 25.02, *p* < .001, η^2^ = .046) (Table 6). No significant differences were observed across age groups, education levels or provincial regions (*p* > .05). Post hoc comparisons indicated that male participants scored significantly lower than female participants (*p* = 0.017). After adjusting for cannabis consumption status, biological sex remained significant.

## Discusssion

Public education about the potential adverse health effects of cannabis plays an important role in supporting informed decision-making in the context of legal cannabis use. Understanding how well adults can access, interpret and apply cannabis-related health information is relevant for identifying potential gaps in public-oriented health education. The CHLQ was developed to assess individual's knowledge and skills related to cannabis health related information. This study aimed to extend prior validation work by examining the CHLQ ‘s reliability and known-groups validity in a Canadian adult sample. While raw CHLQ scores were used for descriptive analyses to summarize performance patterns, Rasch-derived person scores were used to assess group differences, as the scores adjust for item difficulty and relative performance within the sample rather than absolute levels of CHL in the broader Canadian population.

Overall, our findings provide support for the CHLQ's ability to differentiate between relevant subgroups across its four dimensions. Across the sample, notable gaps emerged in knowledge of adverse cannabis health effects, especially among cannabis consumers and non-consumers. Current cannabis consumers generally demonstrated higher performance on the knowledge of cannabis dimension and higher confidence in seeking, accessing and using cannabis health information, potentially reflecting increased familiarity with cannabis products, their labels, retail environments and information sources. However, cannabis consumers expressed lower agreement with risk statements on the CHLQ compared to past or never consumers, a pattern aligning with prior research indicating lower perceived risks among individuals who use cannabis (Leos-Toro et al., 2020a; McKiernan & Fleming, 2017). However, as evidence and public discourse continue to evolve, recent research suggests that perceptions of certain cannabis-related risks have begun to increase over time, despite growing social acceptability of cannabis (Doggett et al., 2025). Variation in risk agreement may reflect differences in experiences, information environments, and familiarity with cannabis. Individuals may be more likely to seek out, trust or recall information that aligns with their existing beliefs or behaviors related to cannabis use (Goodman & Hammond, 2022a; Wickens et al., 2019). From a measurement standpoint, the CHLQ may be sensitive to differences in knowledge and risk perception across sample groups.

Considerable gaps also emerged in respondents’ understanding of harms and risks associated with cannabis. Many respondents, including consumers, were unable to convert or identify THC and CBD concentrations of products, and which ingredients in cannabis are associated with impairment or adverse health effects. This finding aligns broadly with a study that showed that consumers have difficulty interpreting THC and CBD numerical information found on cannabis products such as the amount of THC in each serving sizes and potency level of a cannabis product (Leos-Toro et al., 2020b). This also aligns with national data, where 50% or less of Canadians were able to correctly apply risks to real-life scenarios such as identifying the amount of time they should wait before operating vehicle or heavy machinery after cannabis use (Health Canada, 2024a). Inconsistent product labeling practices, variability in potency and limited public education efforts may contribute to these challenges. To date, there is no universally adopted standardized THC unit and limited evidence defining the threshold of cannabis exposure associated with specific adverse health effects (Hammond, 2021; Wood et al., 2024). However, given that product labeling is a key mechanism for communicating health risks, these findings highlight a potential need for clearer, more accessible labeling practices of cannabis products and continued research to inform evidence-based communication of cannabis health related risks. Our results further illustrate the CHLQ's ability to describe differences between consumers and non-consumers.

Age-related differences were also observed, with younger and middle-aged adults scoring higher than older adults on the CHLQ knowledge-based dimensions. These differences may reflect generational variation in exposure to cannabis-related messaging or lived experiences with cannabis. These findings align with a prior study demonstrating that emerging adults (ages 18–24) are more likely to encounter cannabis advertisements and prevention messages compared to older adults (Huỳnh et al., 2025). Similarly, other research has highlighted the role of both public campaigns and interpersonal communication in shaping cannabis-related perceptions, attitude and behaviours (Schwartz et al., 2024; Tveleneva et al., 2022). The CHLQ's sensitivity to age-related differences provides additional support for its utility in describing CHL across subgroups. These findings also highlight a need for further evaluation, and targeting of cannabis education efforts, especially to older adults, as part of public-oriented health education efforts.

Overall, our findings demonstrate that the CHLQ may serve as a descriptive tool for examining patterns of CHL across different subgroups. Several findings align with a growing body of literature indicating that cannabis literacy remains limited among Canadians, in particular among both recreational and medical cannabis consumers (Goodman & Hammond, 2022b; Kosa et al., 2017). Although early post-legalization campaigns (2018–2019) raised awareness, recent data suggest both exposure and recall of these cannabis related messages have declined (Goodman et al., 2024; Health Canada, 2018b; Leos-Toro et al., 2020a). In the 2024 CCS, among those who consumed cannabis (29%), 63% reported seeing the health warnings, whereas in a separate question assessing recall of broader public education or awareness campaigns, 50% reported not recalling any messaging, which is an increase from 2019. Of those who heard or saw campaigns, 68% believed it was credible information (Health Canada, 2024a). Reduced funding and campaign saturation likely contribute to this decline, underscoring the need for renewed, evidence-informed public health messaging that clearly communicates potential cannabis risks (CHERP, 2023; Health Canada, 2024b). Additionally, ongoing evaluation of CHL within adult populations is needed alongside continued strengthening of the scientific evidence on cannabis-related health effects to support risk education and evaluation of CHL.

Continued refinement of the CHLQ is welcomed, including replication in larger and more diverse samples. Future research should also examine CHL among healthcare providers and retail staff, as these individuals may serve as important sources of information and knowledge translators within communities. Additional work should examine whether self-reported confidence in using cannabis-related health information corresponds to informed decision-making and consumption practices. As the evidence related to cannabis health effects continues to evolve, ongoing evaluation of public health messaging using the CHLQ may help the development of clear, accurate and accessible communication strategies.

### Limitations

Our study has limitations common to cross-sectional, survey-based research and exploratory measurement tool development. First, although the CHLQ has undergone preliminary validation, it remains exploratory; this study contributes incrementally to its refinement. The analyses were conducted using the same dataset as the initial Rasch-based validation of the CHLQ, and thus the present findings should be interpreted as providing incremental evidence of reliability and validity rather than serving an independent replication of the measure. Ongoing work will be continued by the research team to examine the performance of the CHLQ in diverse populations.

Second, our sample may not fully represent the Canadian population, as current cannabis consumers were overrepresented in our sample (42%) compared to the national estimates (∼29%)(Health Canada, 2024c). This oversampling may have influenced overall score distributions and observed group differences for some of the dimensions of the CHLQ, particularly the knowledge of cannabis and information seeking dimensions. Thus, the findings of this study should be interpreted within the context of this particular sample and not generalized to the broader Canadian population.

Third, self-reported data may introduce response bias, with participants potentially overestimating their knowledge or confidence. Lastly, Likert-scale responses were treated as continuous variables in the Rasch analysis. While debates remain about this approach, Rasch modeling supports parametric testing by addressing issues of ordinal scaling and non-uniform response intervals by transforming raw scores into interval data based on person-item probability functions (Boone et al., 2014; Jacques et al., 2025; Medvedev et al., 2020; Mellenbergh & Vijn, 1981; Stone et al., 1999; Tennant & Conaghan, 2007; Wright & Mok, 2004). To reduce bias across demographic subgroups, we performed Differential Item Functioning analysis prior to the group comparisons analysis, enhancing validity and transparency.

## Conclusion

This study provides evidence supporting the CHLQ's reliability and known-groups validity through Rasch-calibrated scales, with moderate to acceptable internal consistency across dimensions. Differences in CHLQ scores were observed by cannabis consumption status age and in some cases, education level. Current cannabis consumers demonstrated higher confidence in using cannabis-related health information but lower agreement with risk-related statements, a pattern broadly consistent with prior literature on cannabis risk perception and suggestive of the CHLQ's sensitivity to nuanced differences across dimensions of CHL. Overall, the findings indicate that the CHLQ can describe variation in CHL within an adult sample and may support further evaluation of subgroups in the context of public-oriented cannabis education. Continued refinement of the CHLQ in independent and more diverse adult samples is welcomed to strengthen its application throughout research.

## Supplemental Material

sj-docx-1-dre-10.1177_00472379261439959 - Supplemental material for The Cannabis Health Literacy Questionnaire – Assessing Reliability and Known-Groups Validity in a Canadian Adult Sample Supplemental material, sj-docx-1-dre-10.1177_00472379261439959 for The Cannabis Health Literacy Questionnaire – Assessing Reliability and Known-Groups Validity in a Canadian Adult Sample by Queen Jacques, Zhiwei Gao, Maisam Najafizada, Lisa Bishop, Rachel Howells and Jennifer Donnan in Journal of Drug Education

sj-docx-2-dre-10.1177_00472379261439959 - Supplemental material for The Cannabis Health Literacy Questionnaire – Assessing Reliability and Known-Groups Validity in a Canadian Adult Sample Supplemental material, sj-docx-2-dre-10.1177_00472379261439959 for The Cannabis Health Literacy Questionnaire – Assessing Reliability and Known-Groups Validity in a Canadian Adult Sample by Queen Jacques, Zhiwei Gao, Maisam Najafizada, Lisa Bishop, Rachel Howells and Jennifer Donnan in Journal of Drug Education

sj-docx-3-dre-10.1177_00472379261439959 - Supplemental material for The Cannabis Health Literacy Questionnaire – Assessing Reliability and Known-Groups Validity in a Canadian Adult Sample Supplemental material, sj-docx-3-dre-10.1177_00472379261439959 for The Cannabis Health Literacy Questionnaire – Assessing Reliability and Known-Groups Validity in a Canadian Adult Sample by Queen Jacques, Zhiwei Gao, Maisam Najafizada, Lisa Bishop, Rachel Howells and Jennifer Donnan in Journal of Drug Education
