# Supplementary material for: The Cannabis Health Literacy Questionnaire – Assessing Reliability and Known-Groups Validity in a Canadian Adult Sample
Source: J Drug Educ. 2026 Apr 8;55(3):152–75. doi: 10.1177/00472379261439959 (PMC13392162; doi:10.1177/00472379261439959)
Supplement: sj-docx-1-dre-10.1177_00472379261439959 - Supplemental material for The Cannabis Health Literacy Questionnaire – Assessing Reliability and Known-Groups Validity in a Canadian Adult Sample [file sj-docx-1-dre-10.1177_00472379261439959.docx]

**Appendix A: Cannabis Health Literacy Survey**

**Eligibility Questions**

1. In what province or territory do you reside in?

- Alberta
- British Columbia
- Manitoba
- New Brunswick
- Newfoundland & Labrador
- Nova Scotia
- Northwest Territories
- Nunavut
- Ontario
- Prince Edward Island
- Quebec
- Saskatchewan
- Yukon
- I do not live in Canada.

1. What is your age?

- Under 19
- 19-29 years
- 30-39 years
- 40-49 years
- 50-59 years
- 60-69 years
- 70 years and older

**EQ5D- Quality of Life Scale**

1. Mobility

- I have no problems in walking about.
- I have slight problems in walking about.
- I have moderate problems in walking about.
- I have severe problems in walking about.
- I am unable to walk about.

1. Self-Care

- I have no problems washing or dressing myself.
- I have slight problems washing or dressing myself.
- I have moderate problems washing or dressing myself.
- I have severe problems washing or dressing myself.
- I am unable to wash or dress myself.

1. Usual Activities (e.g., work, study, housework, family, or leisure activities)

- I have no problem doing my usual activities.
- I have slight problems doing my usual activities.
- I have moderate problems doing my usual activities.
- I have severe problems doing my usual activities.
- I am unable to do my usual activities.

1. Pain/ Discomfort

- I have no pain or discomfort.
- I have slight pain or discomfort.
- I have moderate pain or discomfort.
- I have severe pain or discomfort.
- I have extreme pain or discomfort.

1. Anxiety/Depression

- I am not anxious or depressed.
- I am slightly anxious or depressed.
- I am moderately anxious or depressed.
- I am severely anxious or depressed.
- I am extremely anxious or depressed.

1. We would like to know how good or bad your health is TODAY. This scale is numbered from 0 to 100; 100 means the best health you can imagine and 0 means the worst health you can imagine.

Please use the scale below to indicate how your health is TODAY.

|  | 0 | 10 | 20 | 30 | 40 | 50 | 60 | 70 | 80 | 90 | 100 |
| --- | --- | --- | --- | --- | --- | --- | --- | --- | --- | --- | --- |

| Please use the scale below to indicate how your health is TODAY. | 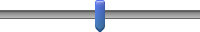 |
| --- | --- |

**Cannabis Health Literacy Questionnaire**

**To what extent do you agree or disagree with the following statements:**

1. I am confident I know where to find information about cannabis.

- Strongly Disagree
- Disagree
- Neither disagree nor agree
- Agree
- Strongly agree

1. I am confident I can ask questions to a health care provider about cannabis.

- Strongly Disagree
- Disagree
- Neither disagree nor agree
- Agree
- Strongly agree

1. I am confident I know where to find information on how to manage unpleasant side effects with cannabis use.

- Strongly Disagree
- Disagree
- Neither disagree nor agree
- Agree
- Strongly agree

1. I am confident in using the cannabis information I find to make cannabis health-related decisions.

- Strongly Disagree
- Disagree
- Neither disagree nor agree
- Agree
- Strongly agree

1. According to the label displayed below, what is the total THC in this product?


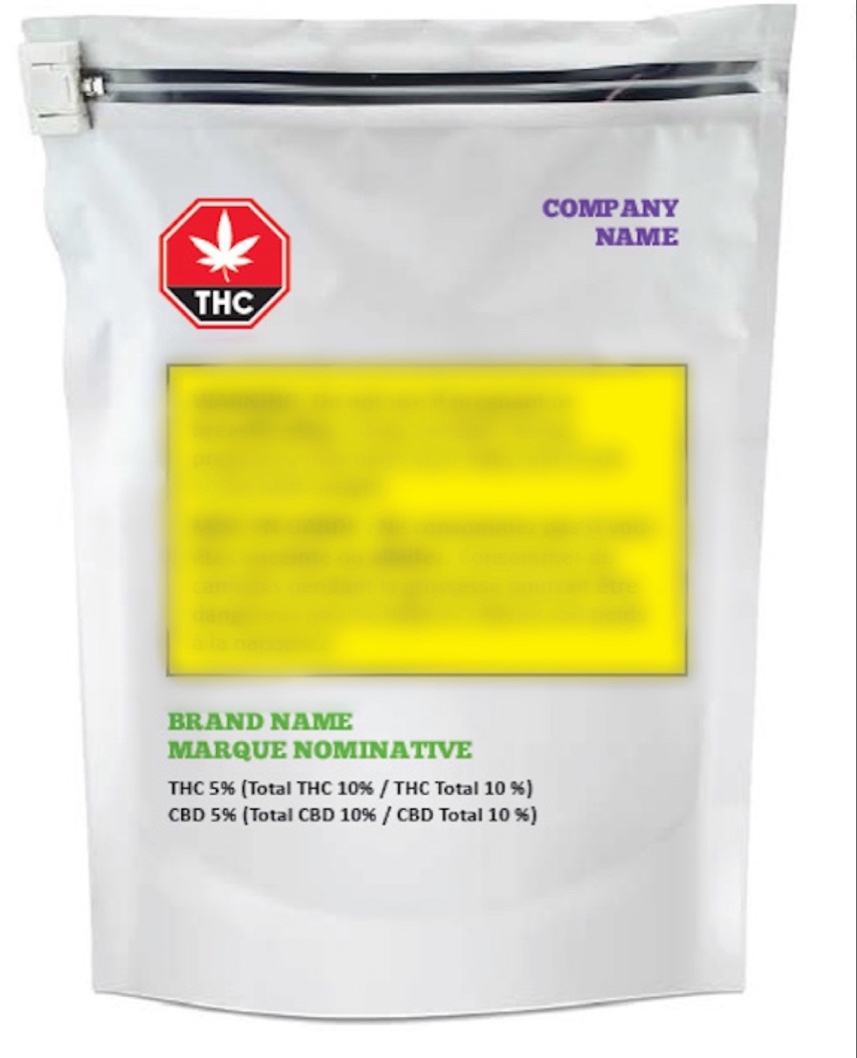


[Image source: Health Canada](https://images.app.goo.gl/ENHUUWtJxRotQQ1F8)

1. 5 %
2. 10%
3. 15%
4. 20%
5. I don’t know
6. According to the product label displayed below, how many milligrams (mg) of cannabinoids are in **one** soft gel?


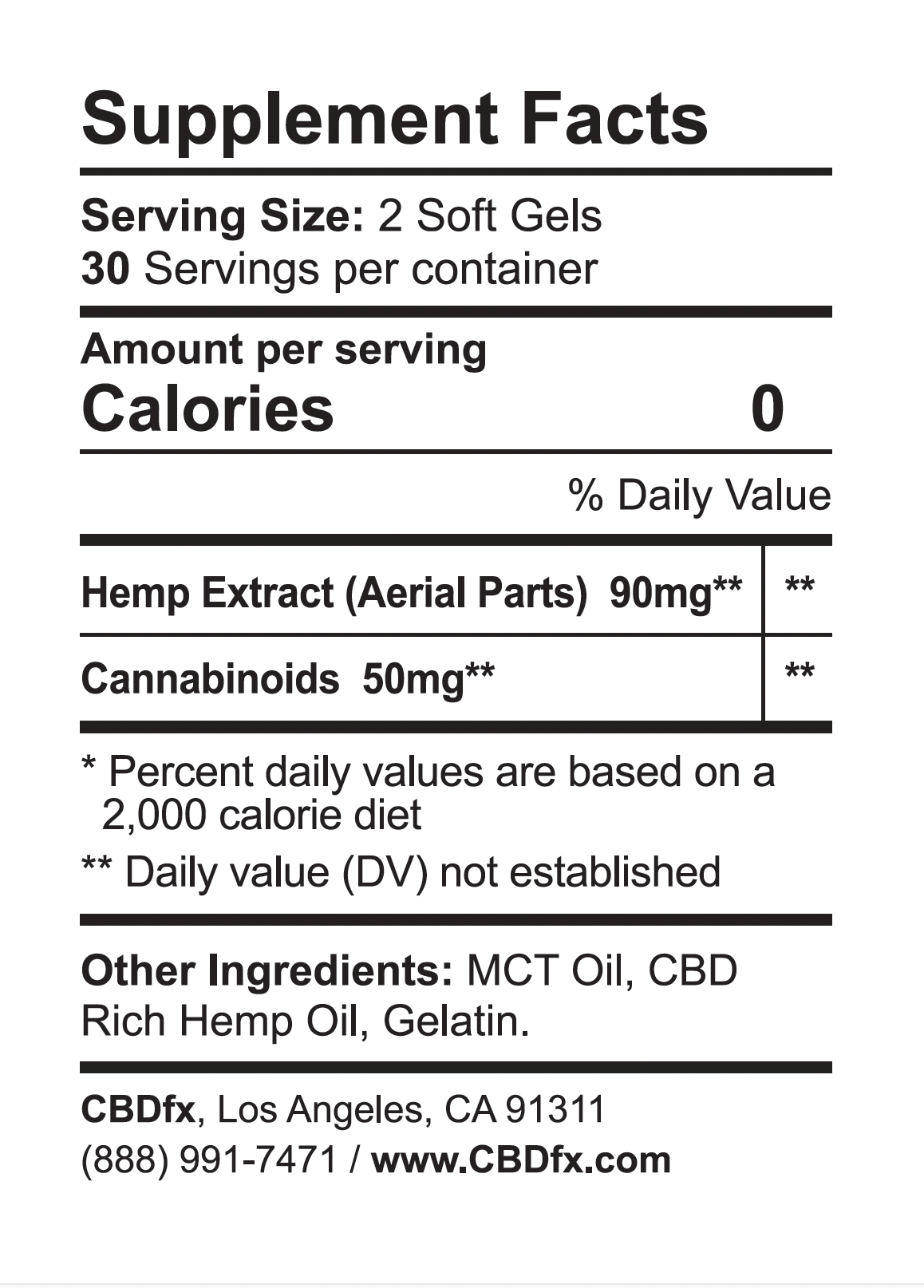


[Image source: CBDFX](https://cbdfx.com/)

1. 15 mg
2. 25 mg
3. 50 mg
4. 100 mg
5. I don’t know
6. If one drop of CBD oil = 1.1mg, how many drops would you need to have 16.5 mg of CBD?
7. 10 drops
8. 15 drops
9. 20 drops
10. 25 drops
11. I don’t know
12. If a syringe of 1 mL has 20 mg of CBD (i.e., 1 mL = 20 mg of CBD), how many mL would you need to have 5 mg of CBD?
13. 0.2 mL
14. 0.25 mL
15. 4 mL
16. 5 mL
17. I don’t know
18. Which of the ingredients of cannabis produces the feeling or experience of being “high”?
19. THC (Tetrahydrocannabinol)
20. CBD (Cannabidiol)
21. CBG (Cannabigerol) & CBN (Cannabinol)
22. Terpenes
23. I don’t know
24. Too much of which ingredient in cannabis products can most likely lead to cannabis poisoning?

Terpenes

CBG (Cannabigerol) & CBN (Cannabinol)

CBD (Cannabidiol)

THC (Tetrahydrocannabinol)

I don’t know.

1. Which method of cannabis consumption typically has the longest delay before experiencing the feeling or experience of being high?
   - - - 1. Eating edibles or drinking cannabis beverages
         2. Lotions or oils for skin
         3. Smoking or vaping
         4. Sprays or Tinctures
         5. I don’t know.
2. Which of the ingredients in cannabis products is most likely to give rise to adverse (i.e., unpleasant) side effects?
3. CBD (Cannabidiol)
4. Terpenes
5. THC (Tetrahydrocannabinol)
6. CBG (Cannabigerol) & CBN (Cannabinol)
7. I don’t know
8. Which of the following health conditions has the least amount of evidence for the medical use of cannabis?
   - - - 1. Epilepsy
         2. Multiple Sclerosis (MS)
         3. Symptoms associated with Cancer
         4. Irritable bowel syndrome (IBS)
         5. I don’t know.
9. If a cannabis flower product contains 300 mg/g of THC, then what is the percentage (%) of THC in the product?
   - - - 1. 0.3%
         2. 3%
         3. 30%
         4. 300%
         5. I don’t know.
10. After smoking cannabis, what is the minimum amount of time a person should wait before driving?
11. less than 1 hour
12. 1 to 3 hours
13. 4 to 8 hours
14. more than 10 hours
15. I don’t know.
16. People can experience harm to brain development from cannabis use when they start consuming cannabis any time before the age of ______.
    - - - 1. 19
          2. 21
          3. 25
          4. 30
          5. I don’t know.

**To what extent do you agree or disagree with the following statements:**

1. Smoking Cannabis can be harmful.

- Strongly Disagree
- Disagree
- Neither disagree nor agree
- Agree
- Strongly agree

1. Using cannabis when pregnant or breastfeeding can be harmful.

- Strongly Disagree
- Disagree
- Neither disagree nor agree
- Agree
- Strongly agree

1. Cannabis can be addictive.

- Strongly Disagree
- Disagree
- Neither disagree nor agree
- Agree
- Strongly agree

1. Driving or operating machinery after cannabis use is dangerous.

- Strongly Disagree
- Disagree
- Neither disagree nor agree
- Agree
- Strongly agree

1. Regular cannabis use can increase the risk for psychosis or schizophrenia.

- Strongly Disagree
- Disagree
- Neither disagree nor agree
- Agree
- Strongly agree

1. Teenagers are at a greater risk of harm from using cannabis than adults.

- Strongly Disagree
- Disagree
- Neither disagree nor agree
- Agree
- Strongly agree

1. **In your opinion, how common are the following side effects with THC consumption?**

|  | Uncommon | Common | I don’t know |
| --- | --- | --- | --- |
| Hallucinations |  |  |  |
| Dry mouth/ red eyes |  |  |  |
| Rapid heart rate |  |  |  |
| Low appetite |  |  |  |

**Cannabis Consumption History**

1. Which of the following best describes your cannabis use history?
   1. I have never consumed cannabis.
   2. I consumed cannabis before legalization and **have continued to consume** cannabis after legalization.
   3. I consumed cannabis before legalization and **stopped, then re-started consuming cannabis** after legalization.
   4. I consumed cannabis before legalization, and I **do not consume cannabis now**
   5. I started consuming cannabis after legalization.
2. Have you consumed cannabis in the past 12 months?

- Yes
- No

1. Which of the following best describes your reason for consuming cannabis?

- Medical purposes (authorized or self-treating)
- Recreational purposes (non-medical)
- Both medical and recreational purposes
- Prefer not to say.

**Demographics**

1. What is the option that best describes your biological sex at birth?

- Male
- Female
- Prefer not to say.

1. What is the option that best describes your current gender identity? (Select all that apply).

- Man
- Woman
- Trans Man
- Trans Woman
- Genderqueer/ Gender non-conforming
- Prefer to self-identify: _______________________________
- Prefer not to say.

1. What is the highest level of education you have completed?

- Did not attend high school.
- Some high school attended.
- High school diploma or equivalent
- Some college/university education attended.
- College diploma or bachelor’s degree
- Graduate degree (e.g., master’s degree, doctoral degree)
- Professional degree (e.g., MD, DDS, DVM, JD, PharmD)

1. Which ethno-racial category best describes you? (Select all that apply)

- Black (e.g., African, Caribbean, African Canadian descent, Caribbean Canadian descent)
- East Asian (e.g., Chinese, Korean, Japanese, Taiwanese descent)
- Latino (e.g., Latin American, Hispanic descent)
- Middle Eastern (e.g., Arab, Persian, West Asian descent, Afghan, Egyptian, Iranian)
- South Asian (e.g., Filipino, Vietnamese, Cambodian, Thai, Southeast Asian descent)
- White (e.g., European descent)
- Mixed Race or Biracial
- Another race category: _______________________
- Prefer not to say.
