# Supplementary material for: The Cannabis Health Literacy Questionnaire – Assessing Reliability and Known-Groups Validity in a Canadian Adult Sample
Source: J Drug Educ. 2026 Apr 8;55(3):152–75. doi: 10.1177/00472379261439959 (PMC13392162; doi:10.1177/00472379261439959)
Supplement: sj-docx-2-dre-10.1177_00472379261439959 - Supplemental material for The Cannabis Health Literacy Questionnaire – Assessing Reliability and Known-Groups Validity in a Canadian Adult Sample [file sj-docx-2-dre-10.1177_00472379261439959.docx]

**Appendix B: Differential Item Functioning Results**

Prior to our analysis, we examined how test items in the CHLQ behave for different groups after accounting for individuals’ abilities. This analysis ensures the fairness of the CHLQ and minimizes test bias, especially given the exploratory nature of our tool. This step was important to confirm that differences in responses reflect actual differences in cannabis health literacy and not in how the questions function across demographics. We conducted differential item functioning (DIF) analyses using the Rasch-Welch test and MH chi square using WINSTEPS software (v.5.3.3.1). Since age and biological sex are the most commonly identified factors influencing questionnaire responses in studies assessing differential item functioning (DIF)(Boone et al., 2014; Bourion-Bédès et al., 2015; Khalaf & Omara, 2022; Yadegari et al., 2019), we focused our DIF analysis on these two variables. Our analysis considered DIF contrast values that were significant (p<0.001) with a moderate to large effect sizes (greater than 0.64).

**Table 1:** DIF for Knowledge of Cannabis dimension by biological sex; a pairwise table was consulted in WINSTEPS

| **Item** | **Group**  **(n)** | **DIF**  **Measure** | **DIF SE** | **Group**  **(n)** | **DIF**  **Measure** | **DIF**  **SE** | **DIF Contrast** | **Chi-square** | **p-value**  **(MH)** |
| --- | --- | --- | --- | --- | --- | --- | --- | --- | --- |
| According to the product label displayed below, how many milligrams (mg) of cannabinoids are in one soft gel? | Male  (500) | 0.05 | 0.11 | Female  (525) | -0.61 | 0.11 | **0.66** | 14.49 | **0.0001** |

p<0.05 (95% confidence); DIF contrast |> 0.64| = moderate to large DIF; Only DIFs with >0.64 reported; MH- Mantel Hanzel

**Result:** This item is significantly easier for females (DIF Measure = -0.61) compared to males (DIF Measure = 0.05). The p-value is significant (p < 0.05), indicating notable DIF, with a moderate effect size of 0.66 (Table 1). No substantial differences in the proportion of gender and biological sex categories were found. No other DIF was found in the CHLQ, thus we proceeded with our regression analyses.

Despite these results, according to Rasch guidelines, the existence of DIF alone does not automatically imply bias in the item; further investigation is needed to understand the reasons behind these observed differences (Boone et al., 2014). We retained the item in our analysis, after conducting a cross-tabulation of the distribution of responses for this item (Table 2)

**Table 2:** Gender and Biological Sex crosstabulation

| **Biological Sex** | | | | |
| --- | --- | --- | --- | --- |
| **Gender** | Male | Female | Prefer not to say | Total |
| Man | 487 | 1 | 0 | 488 |
| Woman | 3 | 517 | 1 | 521 |
| Gender Diverse | 4 | 6 | 1 | 11 |
| Prefer not to say | 6 | 1 | 8 | 15 |

**References**

Boone, W. J., Staver, J. R., & Yale, M. S. (2014). *Rasch Analysis in the Human Sciences*. Springer Netherlands. https://doi.org/10.1007/978-94-007-6857-4

Bourion-Bédès, S., Schwan, R., Laprevote, V., Bédès, A., Bonnet, J.-L., & Baumann, C. (2015). Differential item functioning (DIF) of SF-12 and Q-LES-Q-SF items among french substance users. *Health and Quality of Life Outcomes*, *13*(1), 172. https://doi.org/10.1186/s12955-015-0365-7

Khalaf, M. A., & Omara, E. M. N. (2022). Rasch analysis and differential item functioning of English language anxiety scale (ELAS) across sex in Egyptian context. *BMC Psychology*, *10*(1), 242. https://doi.org/10.1186/s40359-022-00955-w

Yadegari, I., Bohm, E., Ayilara, O. F., Zhang, L., Sawatzky, R., Sajobi, T. T., & Lix, L. M. (2019). Differential item functioning of the SF-12 in a population-based regional joint replacement registry. *Health and Quality of Life Outcomes*, *17*(1), 114. https://doi.org/10.1186/s12955-019-1166-1
