# Supplementary material for: The Cannabis Health Literacy Questionnaire – Assessing Reliability and Known-Groups Validity in a Canadian Adult Sample
Source: J Drug Educ. 2026 Apr 8;55(3):152–75. doi: 10.1177/00472379261439959 (PMC13392162; doi:10.1177/00472379261439959)
Supplement: sj-docx-3-dre-10.1177_00472379261439959 - Supplemental material for The Cannabis Health Literacy Questionnaire – Assessing Reliability and Known-Groups Validity in a Canadian Adult Sample [file sj-docx-3-dre-10.1177_00472379261439959.docx]

**Appendix C**

**Table 1: Significant post hoc pairwise comparisons for the Cannabis Health Literacy Questionnaire dimensions (Tukey HSD)**

| **Knowledge of Cannabis Dimension** | | | | |
| --- | --- | --- | --- | --- |
| **Variables** | **Comparison** | **Mean difference** | **SE** | **p** |
| **Age** | Young adults (18-39) vs older adults (60-70+) | 0.68 | 0.12 | <0.001 |
|  | Middle-aged adults (40-59) vs older adults (60-70+) | 0.44 | 0.13 | 0.002 |
| **Consumer Status** | Current consumer vs past consumer | 0.53 | 0.12 | <0.001 |
|  | Current consumer vs never consumer | 1.20 | 0.11 | <0.001 |
|  | Past consumer vs never consumer | 0.67 | 0.12 | <0.001 |
| **Education level** | Some HS/HS diploma vs graduate degree | -0.60 | 0.21 | 0.023 |
|  | Some HS/HS diploma vs professional degree | -1.16 | 0.30 | <0.001 |
|  | Some HS/HS diploma vs BSc/college | -0.60 | 0.18 | 0.004 |
| **Knowledge of Risks Dimension** | | | | |
| **Consumer Status** | Current consumer vs past consumer | -0.64 | 0.11 | <0.001 |
|  | Current consumer vs never consumer | -1.06 | 0.99 | <0.001 |
|  | Past consumer vs never consumer | -0.42 | 0.11 | <0.001 |
| **Understanding Harms and Risks Dimension** | | | | |
| **Age** | Young adults (18-39) vs older adults (60-70+) | 1.25 | 0.13 | <0.001 |
|  | Young adults (18-39) vs middle-aged adults (40-59) | 0.57 | 0.13 | <0.001 |
|  | Middle-aged adults (40-59) vs older adults (60-70+) | 0.67 | 0.14 | <0.001 |
| **Consumer Status** | Current consumer vs past consumer | 0.63 | 0.13 | <0.001 |
|  | Current consumer vs never consumer | 1.84 | 0.12 | <0.001 |
|  | Past consumer vs never consumer | 1.21 | 0.13 | <0.001 |
| **Seek, Access and Use Cannabis Health Information Dimension** | | | | |
| **Biological Sex** | Male vs female | 0.43 | 0.15 | 0.017 |
| **Consumer Status** | Current consumer vs past consumer | 0.66 | 0.19 | 0.001 |
|  | Current consumer vs never consumer | 1.23 | 0.18 | <0.001 |
|  | Past consumer vs never consumer | 0.57 | 0.20 | 0.010 |

***Note:*** *Positive mean differences indicate higher scores for the first-listed group.*

**Table 2: ANCOVA Results for CHLQ dimensions (Adjusted for Cannabis Use Status)**

| **Source of variance** | **df** | **F** | **p** | η² |
| --- | --- | --- | --- | --- |
|  | *Knowledge of Cannabis Dimension* | | | |
| Age | 2 | 9.07 | **<0.001** | 0.017 |
| Biological Sex | 2 | 0.40 | 0.406 | 0.001 |
| Education level | 3 | 7.05 | **<0.001** | 0.020 |
| Provincial region | 3 | 1.51 | 0.211 | 0.004 |
| Consumer Status |  |  | **<0.001** |  |
|  | *Knowledge of Risks Dimension* | | | |
| Age | 2 | 39.52 | 0.117 | 0.004 |
| Biological Sex | 2 | 0.96 | 0.384 | 0.002 |
| Education level | 3 | 2.60 | 0.051 | 0.008 |
| Provincial region | 3 | 0.42 | 0.739 | 0.001 |
| Consumer Status |  |  | **<0.001** |  |
|  | *Understanding Harms and Risks Dimension* | | | |
| Age | 2 | 33.44 | **<0.001** | 0.061 |
| Biological Sex | 2 | 0.41 | 0.66 | 0.001 |
| Education level | 3 | 0.63 | 0.60 | 0.002 |
| Provincial region | 3 | 2.73 | **0.043** | 0.008 |
| Consumer Status |  |  | **<0.001** |  |
|  | *Seek, Access and Use Cannabis Health Information Dimension* | | | |
| Age | 2 | 2.05 | 0.129 | 0.004 |
| Biological Sex | 2 | 4.40 | **0.012** | 0.008 |
| Education level | 3 | 0.79 | 0.502 | 0.002 |
| Provincial region | 3 | 2.47 | 0.060 | 0.007 |
| Consumer Status |  |  | **<0.001** |  |

**Note.** η² = eta-squared.
